# Bile acid metabolism regulated by the gut microbiota promotes non-alcoholic steatohepatitis-associated hepatocellular carcinoma in mice

**DOI:** 10.18632/oncotarget.24066

**Published:** 2018-01-06

**Authors:** Shoji Yamada, Yoko Takashina, Mitsuhiro Watanabe, Ryogo Nagamine, Yoshimasa Saito, Nobuhiko Kamada, Hidetsugu Saito

**Affiliations:** ^1^ Division of Pharmacotherapeutics, Faculty of Pharmacy, Keio University, Minato-ku, Tokyo 105-8512, Japan; ^2^ Graduate School of Media and Governance, Keio University, Fujisawa, Kanagawa 252-0882, Japan; ^3^ Division of Gastroenterology and Hepatology, Department of Internal Medicine, Keio University, Shinjuku-ku, Tokyo 160-8582, Japan; ^4^ Division of Gastroenterology, Department of Internal Medicine, The University of Michigan Medical School, Ann Arbor, MI, 48109, USA

**Keywords:** deoxycholic acid, bile acid metabolites, mTOR, hepatocellular carcinoma

## Abstract

Gut microbiota plays a significant role in the development of hepatocellular carcinoma (HCC) in non-alcoholic steatohepatitis (NASH). However, understanding of the precise mechanism of this process remains incomplete. A new class steatohepatitis-inducing high-fat diet (HFD), namely STHD-01, can promote the development of HCC without the administration of chemical carcinogens. Using this diet, we comprehensively analyzed changes in the gut microbiota and its metabolic functions during the development of HCC in NASH. Mice fed the STHD-01 developed NASH within 9 weeks. NASH further progressed into HCC by 41 weeks. Treatment with antibiotics significantly attenuated liver pathology and suppressed tumor development, indicating the critical role of the gut microbiota in tumor development in this model. Accumulation of cholesterol and bile acids in the liver and feces increased after feeding the mice with STHD-01. Treatment with antibiotics did not reverse these phenotypes. In contrast, accumulation of secondary bile acids was dramatically reduced after the treatment with antibiotics, suggesting the critical role of the gut microbiota in the conversion of primary bile acids to secondary bile acids. Secondary bile acids such as deoxycholic acid activated the mTOR, pathway in hepatocytes. Activation of mTOR was observed in the liver of mice fed STHD-01, and the activation was reduced when mice were treated with antibiotics. Collectively, bile acid metabolism by the gut microbiota promotes HCC development in STHD-01-induced NASH.

## INTRODUCTION

The number of patients with non-alcoholic fatty liver disease (NAFLD) has been increasing in the recent years [1]. In patients with NAFLD, dysregulation of adipokines, insulin resistance, and dyslipidemia lead to fat accumulation in the liver [2–4]. Activation of Kupffer cells and hepatic stellate cells in NAFLD patients elicits inflammation and fibrogenesis in the liver, leading to the development of non-alcoholic steatohepatitis (NASH). Approximately 10% of NAFLD patients develop NASH. In addition, around 10% of NASH patient further develop liver cirrhosis and hepatocellular carcinoma (HCC) [5–7]. Thus, the prevalence of NASH-associated HCC has been gradually increasing in the recent years [8–11]. Although HCC is the fifth most common malignant tumor in the world, the precise mechanisms by which NASH causes HCC are not entirely understood [1–13].

It has been reported that various kinds of genetic and environmental factors are involved in the development and progression of HCC in NASH. Several animal models of NASH-associated HCC are available to study the mechanisms of carcinogenesis. For example, lack of certain genes such as melanocortin 4 receptor *(Mc4r)* and lacking the tumor suppressor tuberous sclerosis complex (*Ltsc1)* leads to the development of spontaneous HCC in mice fed a high-fat diet (HFD) [12, 13]. Likewise, long-term feeding of mice with HFD can promote the development of HCC in NASH [14, 15]. Although these models are used to investigate the mechanisms of HCC development, they also have limitations. For instance, in humans, individuals who do not show variations in those genes are still able to develop HCC. The latter model requires treatment with carcinogenic chemicals in addition to HFD, such as 7,12-dimethylbenzanthracene (DMBA), to induce HCC development in NASH [14, 16]. Thus, additional animal models that mimic the physiological course of HCC development in humans are essential to fully understand the mechanism of NASH-associated HCC. Increasing evidence has highlighted the critical role of the gut microbiota and its metabolites in the development of HCC in NASH. For example, the gut microbial metabolites promote the secretion of senescence-associated secretory phenotype (SASP) factors that facilitate HCC development in hepatic stellate cells (HSCs) [14]. Lipoteichoic acids, which are components of cell walls of gram-positive commensal bacteria, promote the production of prostaglandin (PG) E_2_ by HSCs. The produced PGE_2_ suppresses the antitumor effect of CD8+ T cells, thereby facilitating liver carcinogenesis [16]. Mice fed the choline-deficient high-fat diet (MCHFD) show increased systemic translocation of lipopolysaccharide (LPS) when treated with dextran sodium sulfate (DSS) [17]. The systemic dissemination of gut-derived LPS leads to liver inflammation and fibrogenesis, eventually causing liver carcinogenesis [17]. On the contrary, administration of probiotic bacteria regulates the immune cell balance in the gut, thus decreasing the risk of liver carcinogenesis [18].

A recent study has reported a new class of HFD, steatohepatitis-inducing HFD (STHD)-01, which contains an increased amount of fatty acids and cholesterol, as compared to commonly used conventional HFDs [19]. It is noteworthy that feeding mice with STHD-01 promotes the development of severe NASH pathology within 9 weeks, while conventional HFD requires more than 24 weeks to develop an equivalent level of NASH [20]. More strikingly, this diet facilitates the progression of HCC from NASH without administration of carcinogenic chemicals [19]. Thus, this new class of HFD can mimic natural course of HCC development in NASH as seen in humans. Hence, in the current study, we use STHD-01 as a model of NASH-associated HCC. By feeding mice this diet, we comprehensively analyzed the alteration of gut microbiota and its metabolites to determine the potential mechanisms by which HFD alone causes NASH-associated HCC.

## RESULTS

### STHD-01 promotes the development of NASH-associated HCC

**Figure 1:**
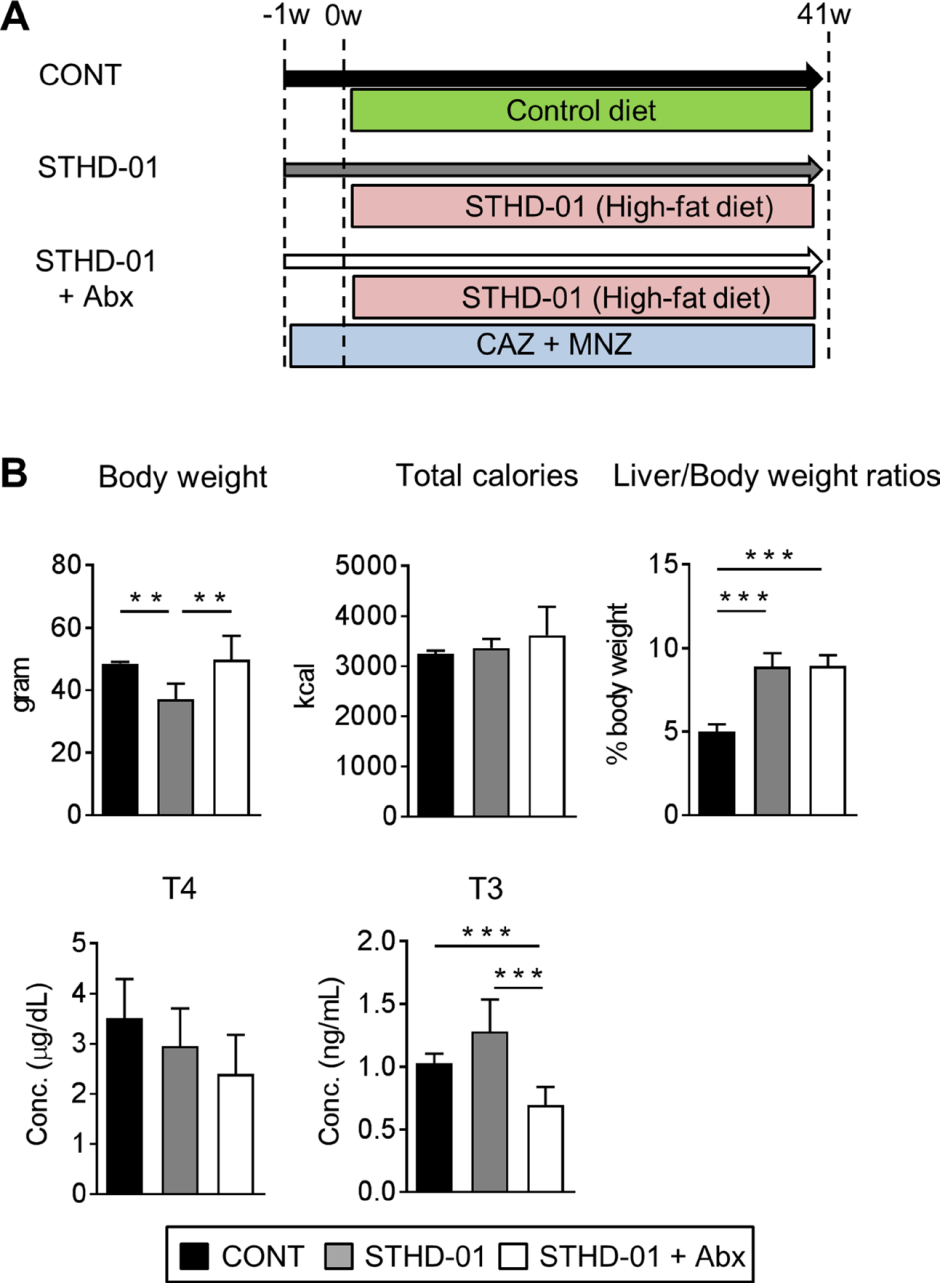
Body characteristics and enzyme-related metabolism changed after feeding of STHD-01 (**A**) Experimental protocol. (**B**) Body weight, total calorie intake, liver/body weight, and plasma triiodothyronine (T3) and thyroxin (T4) levels were measured at 41 weeks post STHD-01 feeding. Data are shown as mean ± SD (CONT, *n* = 5; STHD-01, *n* = 9; STHD-01 + Abx, *n* = 7). ^**^p < 0.01, ^***^ *p* < 0.001 by Tukey’s test.

**Figure 2:**
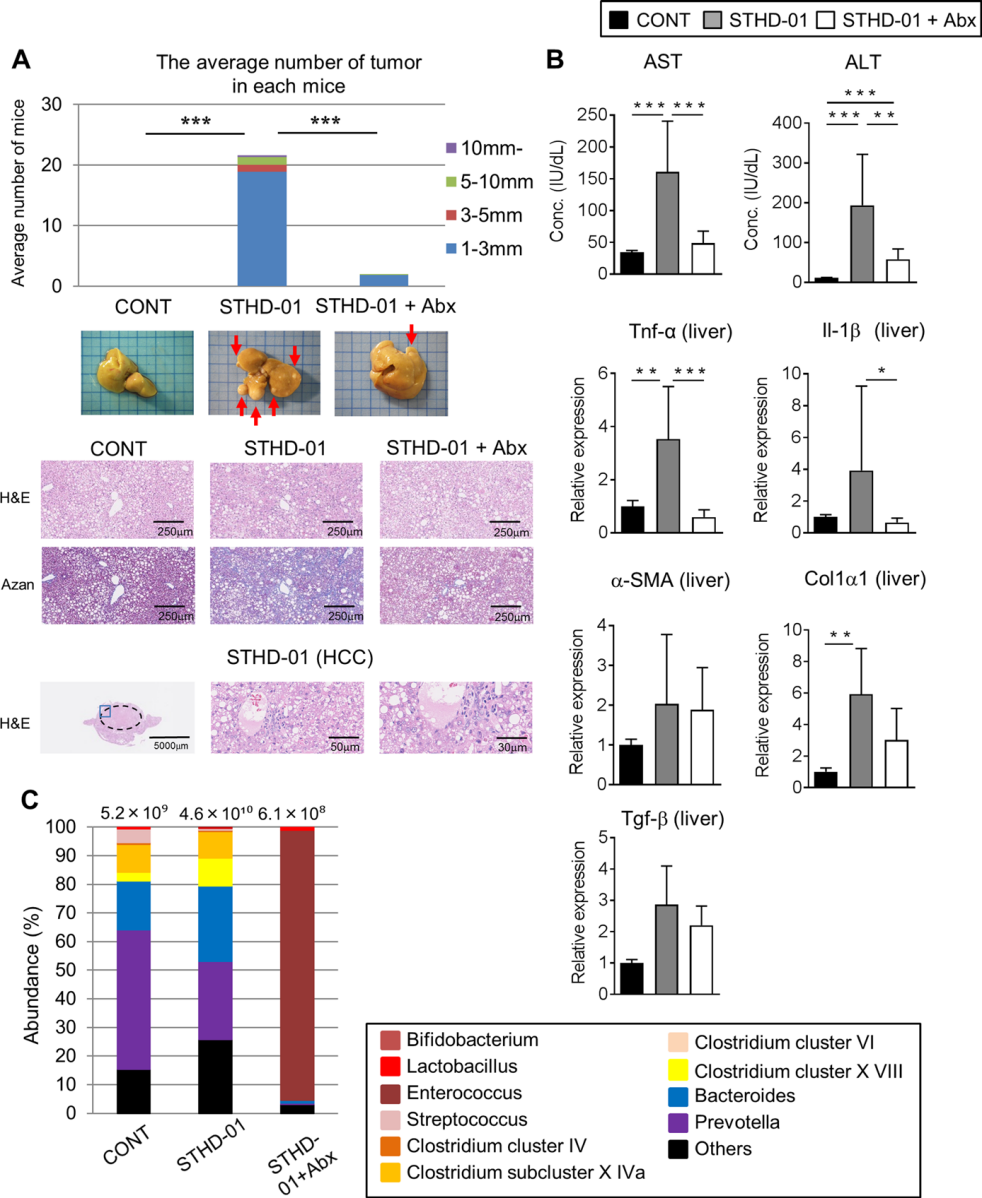
STHD-01 promotes the development of NASH-associated HCC through induction of gut dysbiosis (**A**) The total number of hepatocellular carcinomas (Top), whole liver tissue images (Middle), and representative histology of the liver (Bottom) in each group are shown. (**B**) The plasma level of aspartate aminotransferase (AST) and alanine aminotransferase (ALT) and the mRNA transcription of Tnf-α, Il-1β, α-SMA, Col1α1, Tgf-β in the liver tissue were measured. (A, B) Data are presented as mean ± SD (CONT, *n* = 5; STHD-01, *n* = 9; STHD-01 + Abx, *n* = 7). ^*^p < 0.05, ^**^p < 0.01, ^***^ *p* < 0.001 by Tukey’s test. (**C**) Fecal samples were collected from each individual mouse (CONT, *n* = 5; STHD-01, *n* = 9; STHD-01 + Abx, *n* = 7) at 41 weeks post STHD-01 feeding. Fecal samples within the same group of mice were then pooled and the gut microbiome was analyzed by T-RFLP. The abundance of bacterial genus is shown. The total number of bacteria is given at the top of each column.

SPF C57BL/6J mice were fed a control (CONT) standard (SD) diet (CONT), STHD-01 (STHD-01), or STHD-01 plus antibiotics (Abx; STHD-01+Abx) for 41 weeks (Figure 1A). Total calorie intake between the three groups was not different (Figure 1B). STHD-01-fed mice showed increased liver mass without an increase in the body weight (Figure 1B). Triiodothyronine (T3), a thyroid hormone metabolized from thyroxin (T4), was elevated in the STHD-01 group (Figure 1B). The STHD-01 + Abx group showed increased body weight and T3 levels (Figure 1B). After 41 weeks of STHD-01 feeding, mice developed NASH and HCC (Figure 2A). Liver histology demonstrated a certain degree of age-dependent steatosis in the CONT group (Figure 2A). In STHD-fed mice, there were characteristics of carcinoma and liver dysplasia, such as ballooning, macrophage infiltration, and hepatic fibrogenesis, observed by Azan staining (Figure 2A). Moreover, the STHD-01 group displayed typical features of carcinoma, such as liver cell dysplasia and atypia, and monotonous clear cell changes, (Figure 2A). Notably, the number and size of tumors reduced significantly after treatment with antibiotics, indicating that the gut microbiota contributes to hepatocarcinogenesis in this model. These inflammatory features, caused by STHD-01, were significantly attenuated, and liver tumor development was dramatically attenuated when mice were treated with antibiotics (STHD-01 + Abx group) (Figure 2A). The liver injury markers aspartate aminotransferase (AST) and alanine aminotransferase (ALT), and the transcription of pro-inflammatory cytokines such as tumor nuclear factor (Tnf)-α and interleukin (Il)-1β increased significantly in the STHD-01 group, and these effects were dramatically suppressed by antibiotic treatment (Figure 2B). Upon the feeding of STHD-01, marked infiltration of CD11b^high^ CD11c^low^ inflammatory macrophages was observed in the liver (Supplementary Data 1). Since these cells were significantly decreased by the treatment of antibiotics, macrophage infiltration might contribute to the induction of liver inflammation. The transcription of fibrogenesis markers α-smooth muscle antigen (α-SMA), a1 type 1 collagen (Col1α1), and transforming growth factor **(**Tgf)-β was not altered by administration of antibiotics (Figure 2B). Next, we examined the effect of STHD-01 on the gut microbiota. As shown in Figure 2C, STHD-01 did not affect the total number of bacteria, whereas antibiotic treatment markedly reduced the total number of gut bacteria by more than 100-fold. STHD-01-fed mice showed an increased abundance of *Bacteroides* and *Clostridium* cluster XVIII. In contrast, numbers of *Streptococcus*, *Bifidobacterium*, and *Prevotella* decreased in the mice fed STHD-01 (Figure 2C). In the STHD-01 + Abx group, *Enterococcus* was predominant in the gut microbiota (Figure 2C).

### Bile acid metabolism is altered by STHD-01

**Figure 3:**
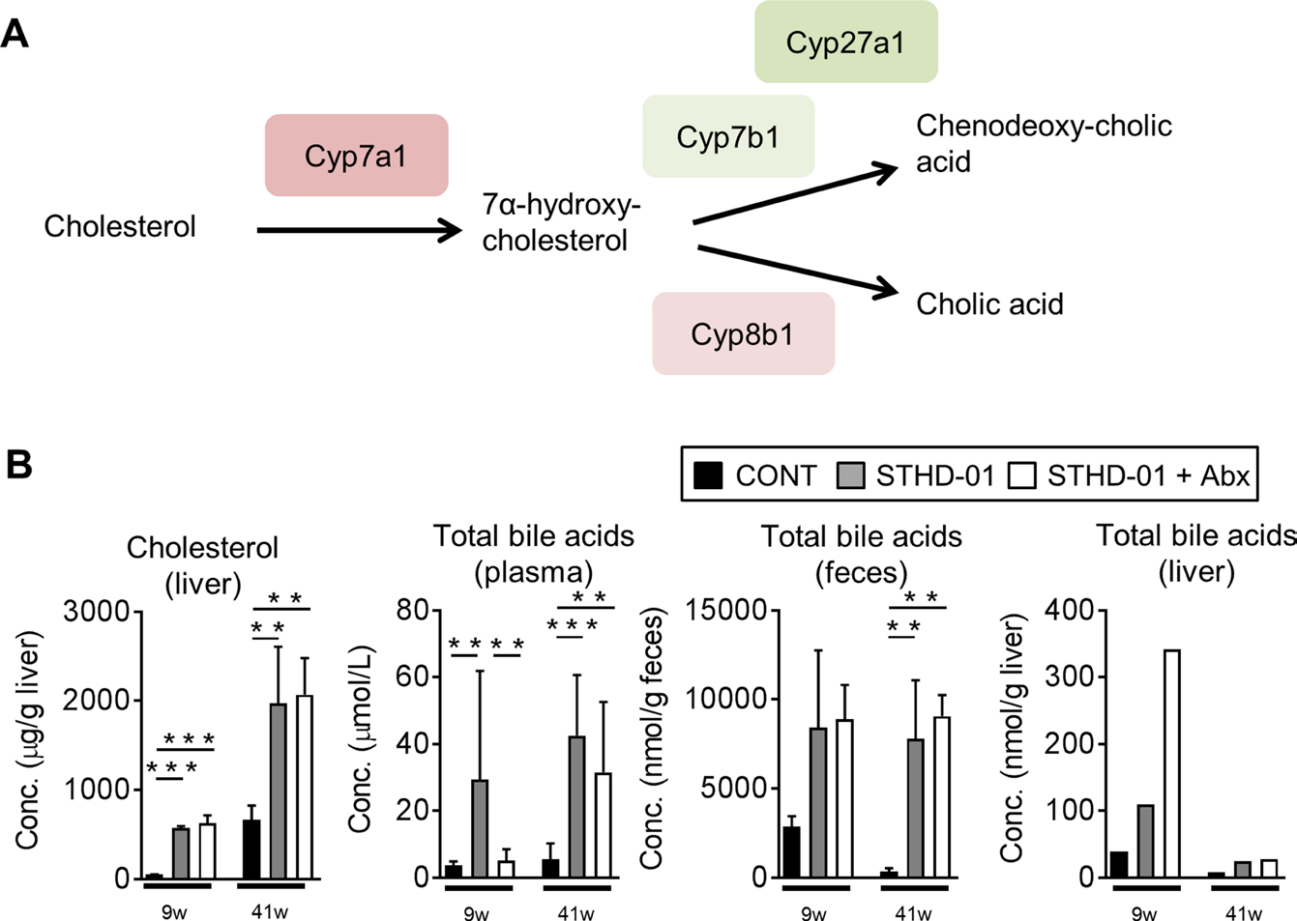
Bile acid synthesis from cholesterol was up-regulated upon the feeding of STHD-01 (**A**) Pathway of bile acid synthesis from cholesterol and related enzymes are shown. (**B**) Total bile acid concentration in plasma, feces, and liver. Liver samples were collected from all mice in each group and pooled. Data are expressed as mean ± SD (CONT, *n* = 5; STHD-01, *n* = 9; STHD-01 + Abx, *n* = 7). ^**^p < 0.01, ^***^p < 0.001 by Tukey’s test.

STHD-01 contains a significant amount of cholesterol, which might influence the development of NASH-associated HCC [19, 20]. Hence, we next examined the extent to which the feeding with STHD-01 leads to the accumulation of cholesterol and its downstream catabolites, bile acids. Figure 3A shows the examined bile acid synthesis pathway, starting from cholesterol. STHD-01 led to an accumulation of cholesterol in the liver after both 9 weeks and 41 weeks after feeding, as expected (Figure 3B). In parallel, STHD-01 led to increased transcription of a bile acid synthesis enzyme, cytochrome P450 (Cyp) 7a1, in the liver (Supplementary Data 2). Other Cyp genes, such as Cyp7b1, Cyp27a1, and Cyp8b1, were not up-regulated upon feeding with STHD-01 (Supplementary Data 2). Consistent with the accumulation of cholesterol in the liver, concentration of total bile acids was significantly elevated in the STHD-01-fed mice in the liver, plasma, and feces (Figure 3B). Depletion of the gut microbiota did not alter the accumulation of cholesterol in the liver (Figure 3B). Moreover, antibiotic treatment did not alter the concentration of total bile acids in the liver and feces, while total the concentration of bile acids in plasma was reduced after depletion of the microbiota in the early phase of NASH (9 weeks) (Figure 3B). These results suggest that STHD-01 causes an accumulation of total bile acids, which is not mediated by the gut microbiota.

### Bile acid reabsorption may be suppressed in HFD-fed mice

**Figure 4:**
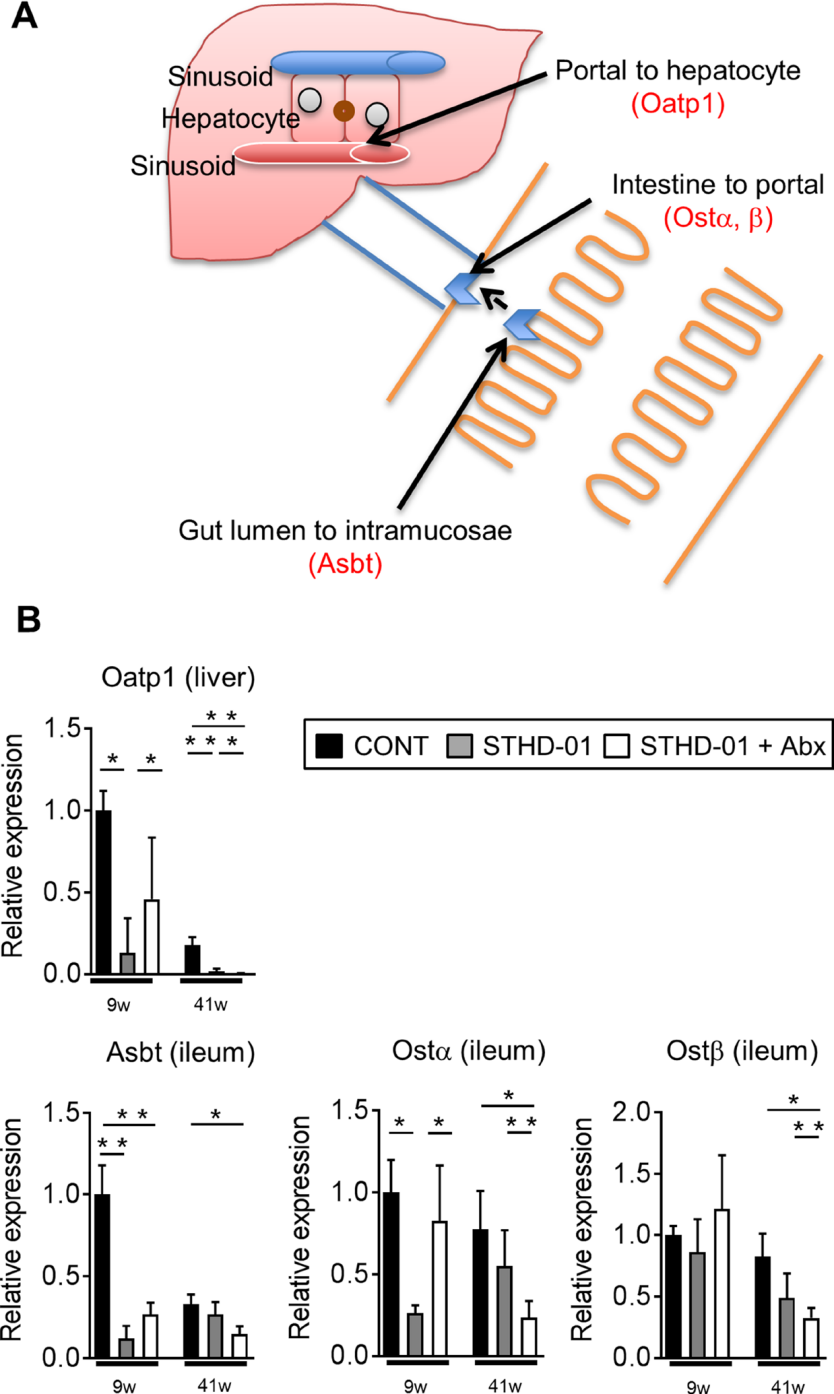
The alternation of bile acids reabsorption transporters by feeding of STHD-01 (**A**) Transporters and transport-proteins related to bile acid secretion/reabsorption into/from the plasma and bile duct are shown. (**B**) The mRNA transcription level of bile acid transporters in the liver and ileum were analyzed. The fold expression relative to 9 week control mice are shown. Data are expressed as mean ± SD (CONT, *n* = 5; STHD-01, *n* = 9; STHD-01 + Abx, *n* = 7). ^*^p < 0.05, ^**^p < 0.01 by Tukey’s test.

To address the mechanisms by which bile acids accumulate in the liver upon consumption of STHD-01, we next examined the effect of STHD-01 on the expression of transporters that are related to bile acid extraction and reabsorption (Figure 4A). At 9 weeks post STHD-01 feeding, transcription of bile acid reabsorption transporters in the ileum and liver, such as sodium-dependent bile acid transporter (Asbt), organic solute transporter (Ost) α and organic anion-transporting polypeptide (Oatp)1, was significantly suppressed in the STHD-01 group, as compared to that in the control group (Figure 4B). The transcription levels of these transporters were restored by depletion of the gut microbiota (Figure 4B). However, at 41 weeks post STHD-01 feeding, transcription of these transporters decreased even in the STHD-01 + Abx group (Figure 4B).

### Bile acid composition is altered by the depletion of gut microbiota

**Figure 5:**
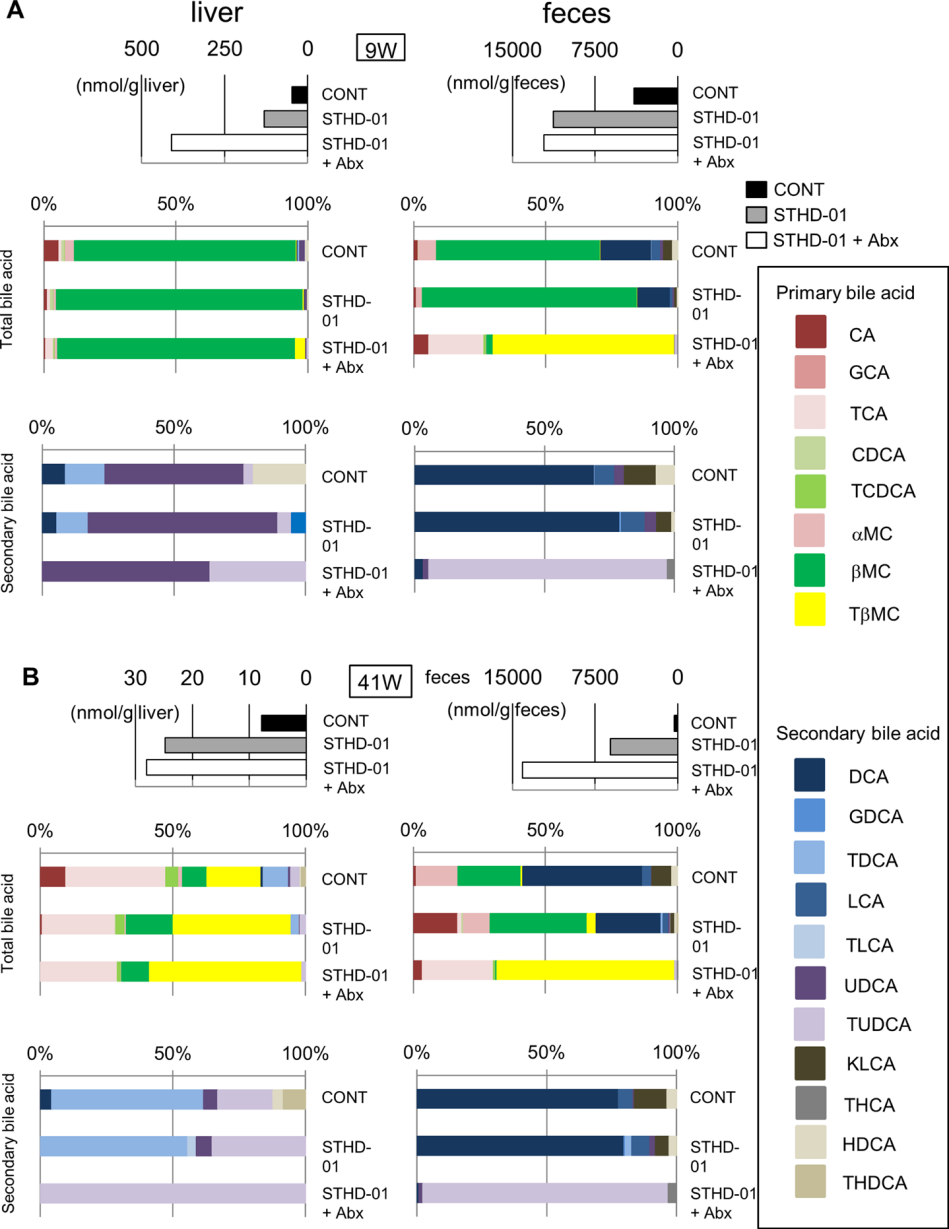
Changes in the composition of bile acids in the liver and feces after feeding of STHD-01 The composition of bile acids in the liver (left) and feces (right) at 9 weeks (**A**) and 41 weeks (**B**) post STHD-01 feeding. Top panel shows the concentration of the total bile acid in each group. Middle panel shows the percent abundance of different primary and secondary bile acids within the total bile acid. Bottom panel shows the percent abundance of different secondary bile acids within the whole pool of secondary bile acids. Sample from all the mice in the same group were pooled and analyzed.

Figure 3 demonstrates the accumulation of cholesterol and total bile acids in the liver upon consumption of STHD-01. Accumulation of total bile acids was not improved by treatment with antibiotics, whereas STHD-01-promoted NASH and HCC were significantly suppressed by the depletion of gut microbiota. These results suggest a possibility that gut microbiota mediate compositional changes in bile acids, rather than promoting their synthesis and reabsorption. Therefore, we next analyzed the composition of bile acids accumulated in the liver and feces. After both 9 weeks and 41 weeks of STHD-01 feeding, primary bile acids were predominant in the liver and the feces (Figure 5A, 5B). At 9 weeks, β-muricholic acid (βMC) was the predominant bile acid in the liver and feces of both control and STHD-01-fed mice (Figure 5A). Treatment with antibiotics decreased the concentration of βMC and increased taurin-conjugated βMC (TβMC) in the feces, but no changes were observed in their levels in the liver (Figure 5A), suggesting that bacteria that mediate the deconjugation of taurine in the gut were depleted by antibiotics. At 41 weeks, TβMC became the predominant primary bile acid in the liver in all three groups of mice, whereas βMC was the predominant primary bile acid in the feces, except in the Abx group (Figure 5B). The concentration of secondary bile acids was lower than that of primary bile acids in feces and almost undetectable in the liver at 9 weeks (Figure 5A). At 9 weeks, secondary bile acids in feces were slightly increased in STHD-01-fed mice than in the control mice, and their levels increased further at 41 weeks (Figure 5A, 5B). Secondary bile acids in feces decreased dramatically when the gut microbiota was depleted by antibiotics (Figure 5A, 5B), indicating that the gut microbiota mediates the conversion of bile acids from primary to secondary.

### Gut microbiota regulates the accumulation of secondary bile acids in the liver

**Figure 6:**
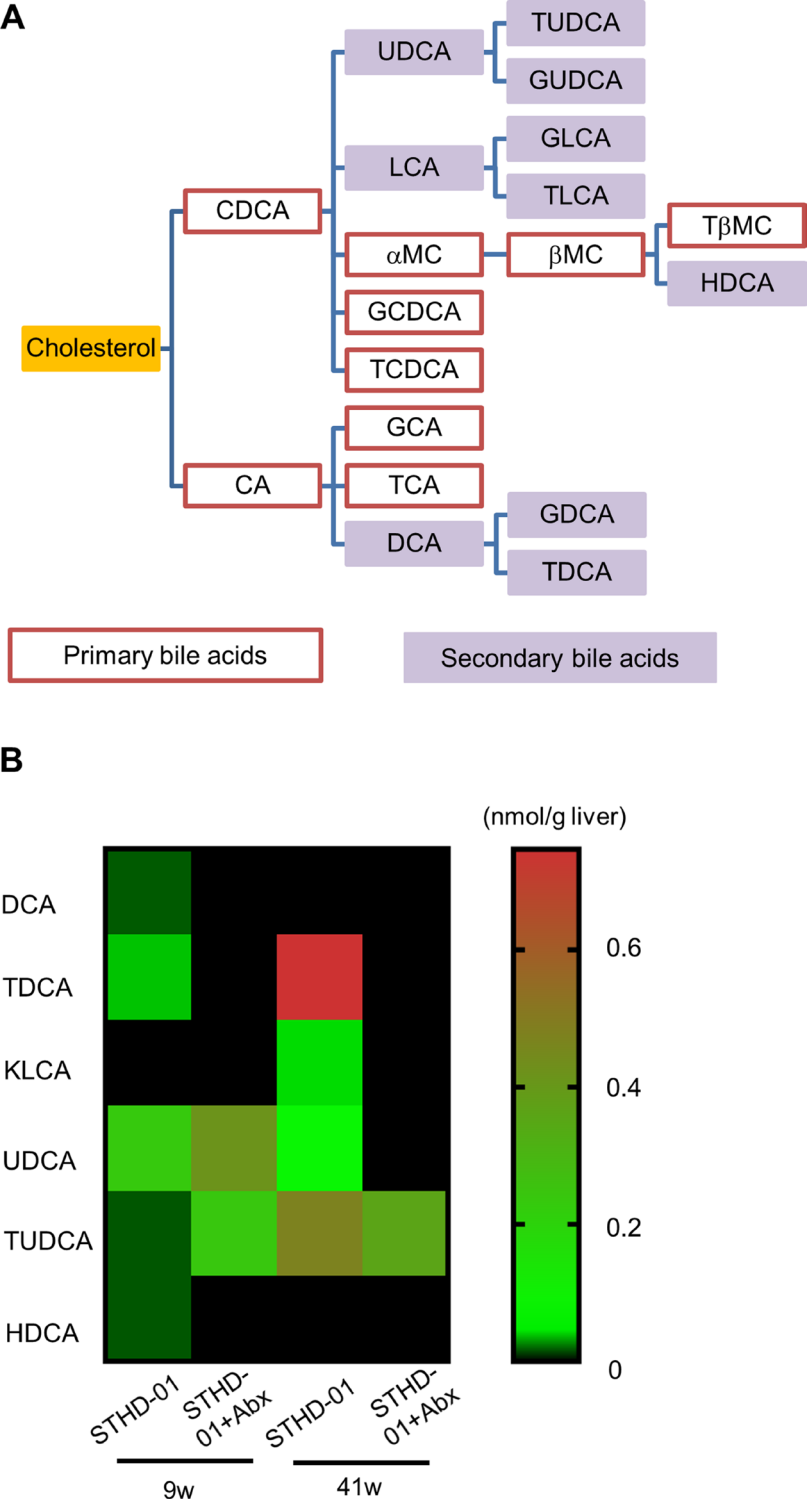
Abundance of secondary bile acids in the liver was regulated by the gut microbiota (**A**) Bile acid metabolic pathways. (**B**) A heat-map of the abundance of detected secondary bile acids in the liver. Sample from all the mice in the same group were pooled and analyzed.

Because the generation of secondary bile acids is mediated by the microbiota in the gut, we further assessed the accumulation of secondary bile acids in the liver of mice developing NASH-associated HCC. Figure 6A shows the metabolic pathway of cholesterol to bile acids. Red line columns indicate primary bile acids and purple columns indicate secondary bile acids. Figure 6B demonstrates the concentration of secondary bile acids in the liver of mice in the STHD-01 and STHD-01 + Abx groups. At 9 weeks, accumulation of deoxycholic acid (DCA), Tauro-DCA (TDCA), and Hyo-DCA (HDCA) induced by STHD-01 was markedly reduced by treatment with antibiotics (Figure 6B). The levels of ursodeoxycholic acid (UDCA) and Tauro-UDCA (TUDCA) in the liver were not different between STHD-01 and STHD-01 + Abx mice (Figure 6B). 12-Keto litho cholic acid (KLCA) was almost undetectable in both groups (Figure 6B). At 41 weeks, TDCA, KLCA, UDCA, and TUDCA accumulated in the liver, and the accumulation was reversed by depletion of the gut microbiota (Figure 6B). DCA and HDCA became undetectable in the liver in the later phase of the disease (Figure 6B). This result suggests that generation of secondary bile acids in the gut and their accumulation in the liver were mediated by the gut microbiota.

### Secondary bile acids activate mTOR signaling in hepatocytes

**Figure 7:**
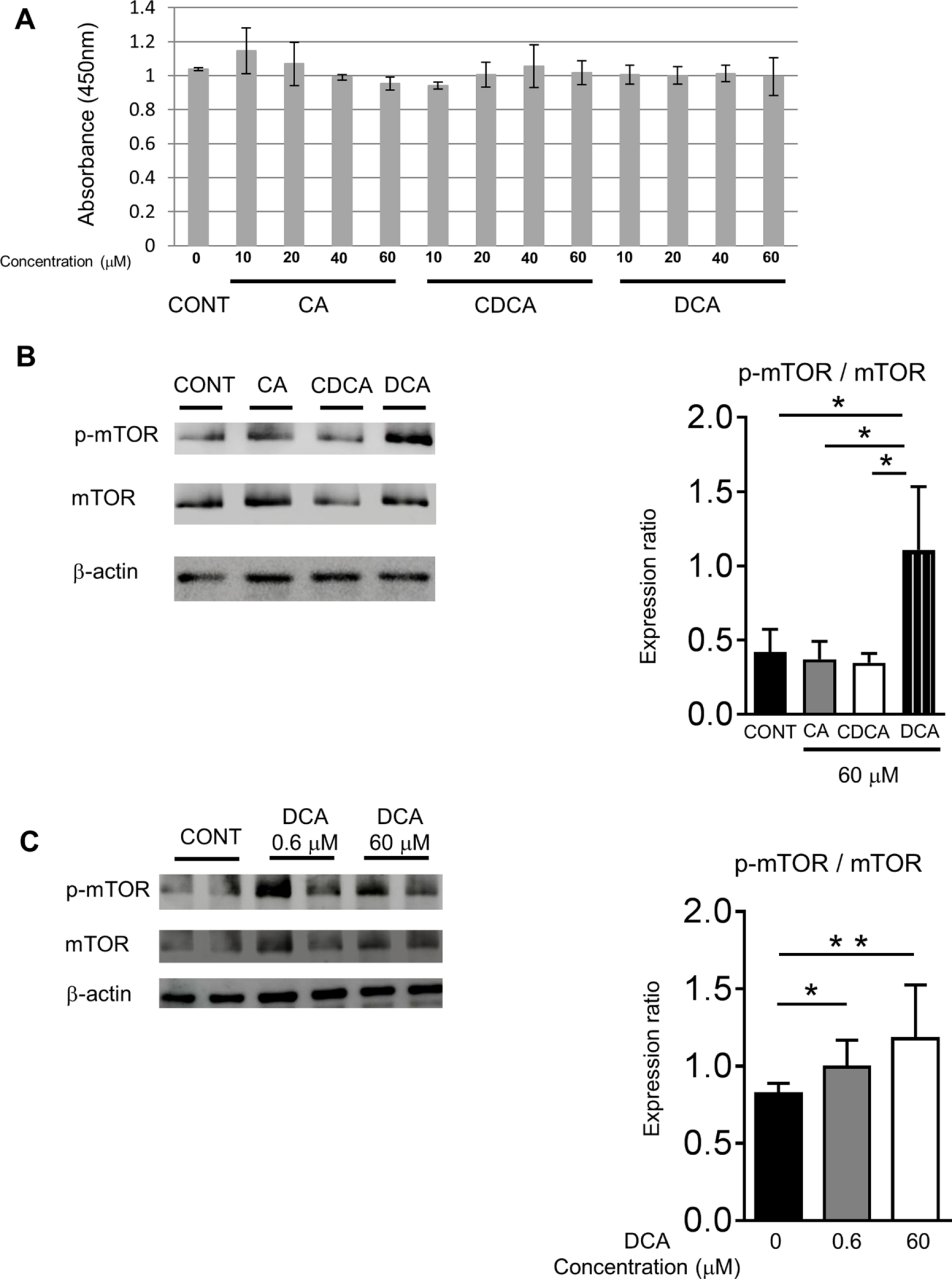
DCA activated the mTOR signaling in the HepG2 cells (**A**) Viability of HepG2 cells after stimulation with primary and secondary bile acids (*n* = 3). (**B**) Phosphorylation of mTOR in HepG2 cells after the stimulation by primary and secondary bile acids (CA, CDCA n = 3; DCA *n* = 5). (**C**) Phosphorylation of mTOR in HepG2 cells after the stimulation by the different concentrations of DCA (*n* = 5). Data are presented as mean ± SD. ^*^p < 0.05 ^**^p < 0.01 by the Tukey’s test.

Our results indicated that STHD-01 promotes the accumulation of secondary bile acids in the liver, resulting in the progression of HCC in NASH. To address the mechanisms by which accumulated secondary bile acids promote the liver carcinogenesis, we stimulated a hepatocyte cell line HepG2 by primary bile acids cholic acid (CA) and chenodeoxycholic acid (CDCA) or a secondary bile acid DCA. Both primary and secondary bile acids did not show any cytotoxicity on HepG2 cells (Figure 7A). Next, we examined the activation of the mammalian target of rapamycin (mTOR) pathway, which is known to be associated with liver carcinogenesis [12]. As shown in Figure 7B, DCA, but not CA and CDCA, induced phosphorylation of mTOR in HepG2 cells. This result suggests that accumulation of secondary bile acids in the liver may promote liver carcinogenesis via activation of the mTOR pathway in hepatocytes. Of note, DCA could induce mTOR activation in HepG2 cells in a lower concentration (0.6 µM), which might be closer to the physiological concentration in the liver (Figure 7C).

### STHD-01 induces mTOR activation in the liver

**Figure 8:**
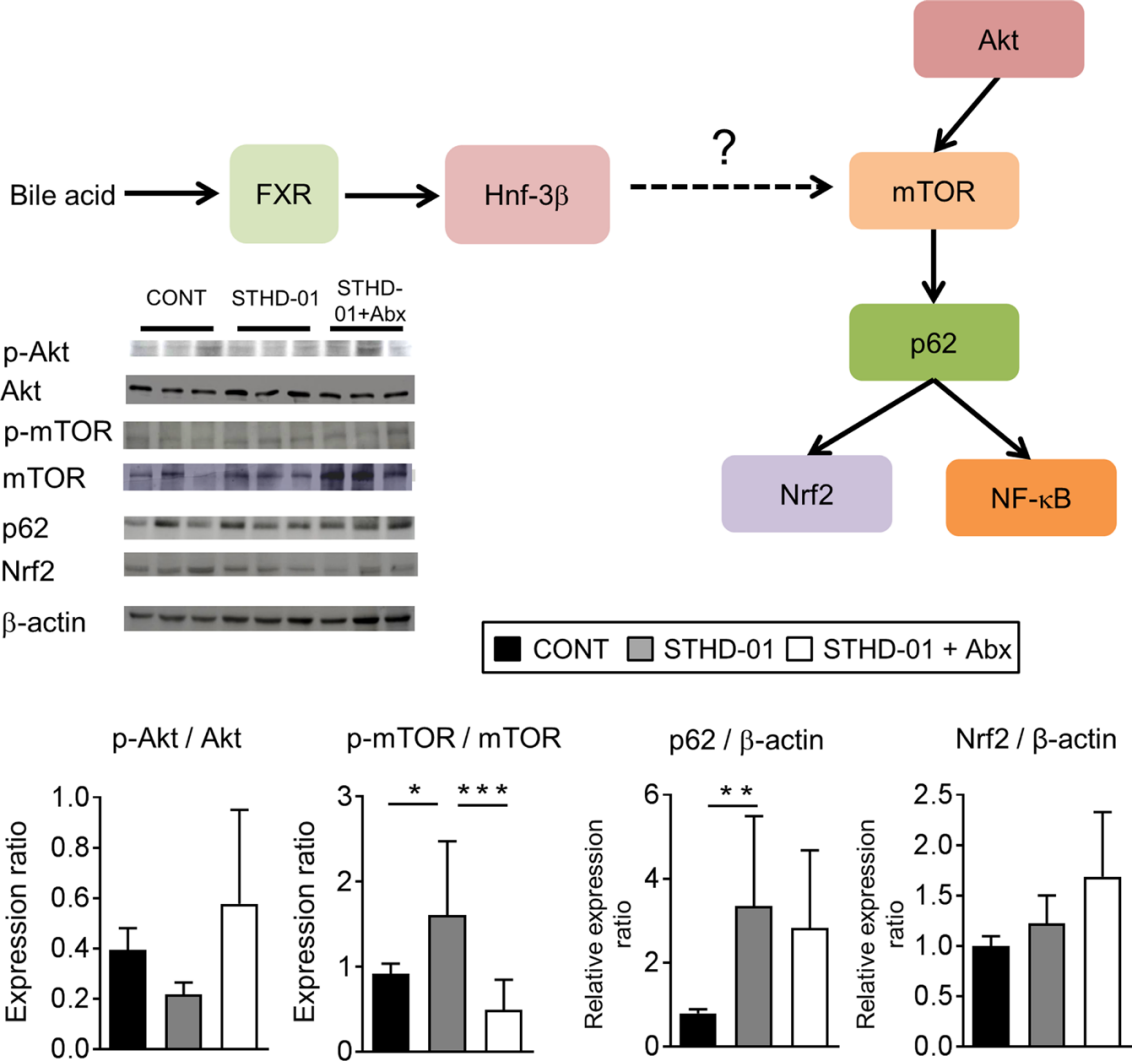
Elevated mTOR activation in the liver in mice fed the STHD-01 (Top) The liver was harvested from mice at 41 weeks post STHD-01 feeding. The activation of mTOR-related pathways was analyzed by western blotting (CONT, *n* = 16; STHD-01, *n* = 16; STHD-01 + Abx, *n* = 7). The representative blots are shown. (Bottom) The quantification of western blot analysis. Data are presented as mean ± SD (CONT, *n* = 16; STHD-01, *n* = 16; STHD-01 + Abx, *n* = 7). ^*^p < 0.05, ^**^ p < 0.01 , ^***^p < 0.001 by Tukey’s test.

Lastly, we assessed the activation of the mTOR pathway in STHD-01-fed mice. We examined the activation of the PI3K- Akt/protein kinase B (Akt) pathway, which is known to lead to mTOR activation, and nucleoporin p62 (p62) and nuclear factor (erythroid-derived 2)-like (Nrf) 2, which are known targets of mTOR [21] in the liver (Figure 8). Phosphorylation of mTOR was significantly elevated in mice fed the STHD-01 compared to control mice and the activation was suppressed by the treatment with antibiotics (Figure 8, Supplementary Data 3). Increased activation of Akt, p62, or Nrf2 was not observed in STHD-01-fed mice (Figure 8). These results suggested that STHD-01 induces the activation of mTOR in the liver, probably mediated by the metabolites generated by the gut microbiota, such as secondary bile acids.

## DISCUSSION

The role of the gut microbiota in the pathogenesis of liver diseases has been repeatedly proposed in recent years. Particularly, the gut microbial metabolites play significant roles in the development of NASH and NASH-associated HCC. Recently, Yoshimoto et al. reported that an HFD diet, along with the administration of a chemical carcinogen DMBA, results in the development of HCC in mice [14]. This phenotype is mediated by the gut microbiota, as treatment with antibiotics has been shown to suppress the development of HCC [14]. Moreover, HFD-induced HCC requires the co-administration of chemical substances. However, since the direct effect of these chemical substances on lipid accumulation or on the composition and/or function of the gut microbiota remains uncertain, the use of these chemical carcinogens might have potential pitfalls and therefore cannot completely represent the natural course of HCC development from NASH in humans. In contrast, a newly developed HFD, STHD-01, has been reported to promote HCC development without co-administration of carcinogenic chemicals [19]. Hence, in the present study, we utilized this clinically relevant HFD, STHD-01, and analyzed the metabolites of gut microbiota in the promotion of oncogenic pathways. We found that the gut microbiota is required for deconjugation of bile acids, as well as for the conversion of primary bile acids to secondary bile acids in STHD-01-fed animals. Therefore, depletion of the gut microbiota by treatment with antibiotics markedly reduced the concentration of secondary bile acids in both feces and liver. Since secondary bile acids might have potential to induce carcinogenic signaling, such as mTOR activation, their metabolism by the gut microbiota play a key role in the development of HCC in STHD-01-fed mice. Notably, secondary bile acids alone are probably not enough to induce the development of HCC since they are already present at significant concentration in the livers of control mice. Since STHD-01 induces pro-inflammatory changes, such as the elevated expression of Tnf-α and Il-1β and the infiltration of inflammatory macrophages, in the liver (Figure 2B and Supplementary Data 1), liver inflammation might augments the secondary bile acid-induced carcinogenesis in the liver.

Gut microbiome analysis revealed that abundance of bacterial genera of *Bacteroides* and *Clostridium* cluster XVIII increased, and that of *Streptococcus*, *Bifidobacterium*, and *Prevotella* decreased after feeding with STHD-01. *Clostridium* is known to metabolize the primary bile acids and convert them to secondary bile acids [14]. Therefore, increased number of these bacteria may correlate with increased accumulation of secondary bile acids in STHD-01-fed mice. *Bacteroides* might contribute to deconjugation of bile acids, which is also required for generation of secondary bile acids [22]. After treatment with antibiotics, numbers of these bacteria were dramatically decreased in the gut. In parallel, generation of secondary bile acids was markedly suppressed in STHD-01 + Abx mice. Notably, accumulation of cholesterol in the liver and total bile acids in the liver, plasma, and feces induced by STHD-01-feeding was not changed by depletion of the gut microbiota. These phenotypes were also seen in mice fed a conventional HFD in a previous report [23]. These results indicate that the gut microbiota does not affect the absorption of cholesterol, primary bile synthesis, and reabsorption of generated bile acids. On the contrary, expression of a bile acid synthesis enzyme, Cyp7a1, was up-regulated in STHD-01-fed mice, but the expression was significantly suppressed by depletion of the microbiota. The concentration of other bile acid synthesis enzymes, such as Cyp7b1, was not changed in Abx-treated mice, as compared to STHD-01-fed mice. Thus, we conclude that the synthesis of bile acids was not changed in these two groups. It is noteworthy that the accumulation of bile acids in the liver was elevated in 9 weeks, but decreased in 41 weeks. This indicates that the excess bile acids of the liver may have overflowed from the damaged liver in the later phase of NASH. Another possibility is that the reabsorption of bile acids from the ileum is impaired in the later phase of NASH. This hypothesis is supported by the bile acid transporter expression in the ileum and liver (Figure 4B). It remains unclear why reabsorption of bile acids is suppressed in the later phase of NASH. One possible explanation is a feedback regulation mechanism that limits the absorption of harmful secondary bile acids.

The precise mechanisms by which secondary bile acids initiate the oncogenic process in the liver remain poorly addressed. Activation of the mTOR pathway is known to be associated with oncogenesis in various types of cancers, such as human cervical and colon cancers [24, 25]. However, the involvement of mTOR activation in liver cancer remains incompletely understood. It has been reported that stimulation of a nuclear receptor farnesoid X receptor (FXR) leads to activation of mTOR pathways [21]. Since bile acids are known ligands for FXR, it is plausible that accumulated bile acids initiate the activation of the oncogenic mTOR pathway in the liver. In accordance with our hypothesis, DCA, but not CA or CDCA, induced activation of mTOR in hepatocytes (Figure 7B, 7C). Since mTOR was activated by DCA, but not by other potential FXR agonists CA and CDCA, this activation might not be mediated through FXR [26, 27] (Figure 7B, 7C and Figure 8). Although the present study suggests an importance of mTOR pathway activation by secondary bile acids in hepatocarcinogenesis, there are other possible mechanisms that might be involved in the promotion of HCC by accumulated secondary bile acids. For example, we found that STHD-01-fed mice displayed an increased expression of pro-inflammatory cytokines in the liver, and inflammation was reduced upon treatment with antibiotics. Since inflammation promotes carcinogenesis in different kinds of cancer, including HCC and secondary bile acids elicit inflammation in hepatocytes [14], accumulated secondary bile acids might contribute to enhanced carcinogenesis through the facilitation of liver inflammation [28]. Moreover, it is also possible that accumulated secondary bile acids activate FXR signaling, thereby promoting tumor development. This notion is supported by the evidence that TβMC, an antagonist of FXR, was elevated in the liver and feces of the STHD-01 + Abx group. Increased TβMC might prevent FXR activation and subsequent tumorigenesis induced by secondary bile acids [29]. In conclusion, mice fed a new class high-fat diet STHD-01 develop HCC in NASH. HCC development is mediated by metabolites produced by the gut microbiota, such as secondary bile acids. Secondary bile acids such as DCA might contribute to oncogenesis, possibly via the activation of mTOR signaling in hepatocytes.

## MATERIALS AND METHODS

### Ethics statement

The use of non-human primates in research. All animal experiments were conducted in accordance with the Institutional Guidelines on Animal Experimentation at Keio University http://www.animal.med.keio.ac.jp/img/kitei.pdf and were approved by The Keio University Institutional Animal Care and Use Committee (Permission #13042-(2)). Our animal care and protocols complied with the national guidelines defined by the Science Council of Japan, available at http://www.scj.go.jp/ja/info/kohyo/pdf/kohyo-20-k16-2e.pdf.

EA Pharma Co., Ltd, which is involved in the research and development, manufacturing, and sales of pharmaceuticals was provided the diet of AIN-93G and STHD-01 for all terms. However, with regards to the present manuscript, the authors state that there are no financial, personal, or professional competing interests that might have influenced the performance or presentation of the work in this manuscript.

### Animals

SPF C57BL/6J mice were fed a conventional CE-2 diet (CLEA Japan Inc., Tokyo, Japan) until the start of the experiments (8 weeks of age). The gut microbiota was normalized by mixing beddings between cages every 2–3 days for 2 weeks. Mice were fed an STHD-01 (11% kcal/protein, 72% kcal/fat, and 17% kcal/nitrogen-free extracts; EA Pharmaceuticals Co., Ltd., Tokyo, Japan) [19] for 9 weeks (short-term experiment) or 41 weeks (long-term experiment). The control group was fed an SD diet (AIN-93G; 19% kcal/protein, 12% kcal/fat, and 69% kcal/nitrogen-free extract). To deplete the gut microbiota, a cocktail of Abx (1 g/L ceftazidime and 1 g/L metronidazole; both from Sigma-Aldrich, Tokyo, Japan) was administered one week before starting STHD-01 feeding. Mice were euthanized after 9 weeks or 41 weeks, and feces, plasma, liver, and small intestinal samples were harvested. Tumor count in the liver tissue and histological evaluation was performed in a blinded manner by two pathologists and a hepatologist from Keio University Hospital and biochemical examinations were conducted according to a previously described method [30].

### Measurement of biomarkers for liver inflammation

Levels of T3 and T4 were analyzed using enzyme-linked immunosorbent assay kits (Alpha Diagnostic Intl. Inc., Antonio, TX). Levels of AST and ALT were measured using Spotchem EZ (SP-4430, Arkray USA Inc., Minneapolis, MN).

### Liver cell preparation

The livers were perfused through the portal vein with FACS buffer [1 g bovine serum albumin (BSA) in 500 mL PBS] and then minced well. The filtrate was centrifuged at 50 g for 1 min, and the supernatant was washed once. The cells were suspended in Histopaque solution (Sigma-Aldrich) and overlaid on an HBSS solution to distinguish monocytes. After centrifugation at 2000 rpm for 20 min, the cells were collected from the upper phase of the Histopaque, and were washed and resuspended in a RPMI1640 medium. After blocking with anti-FcR (CD16/32, BD bioscience, NJ) for 20 min, the cells were incubated with specific monoclonal antibodies at 4°C for 30 min. Liver mononuclear cells were stained with PE-Cy7-conjugated anti-mouse CD11c mAb and fluorescein isothiocyanate (FITC)-conjugated anti-mouse CD11b mAb (both from BD biosciences) were used for analyzing plasmacytoid dendritic cells (pDCs), conventional dendritic cells (cDCs), and macrophages. Background fluorescence was assessed by staining with the relevant isotype control Abs. Stained cells were analyzed by flow cytometry (FACS Cant II, Becton Dickinson Co. Franklin Lakes, NJ), and data were analyzed using the FlowJo software (FlowJo, LLC, Ashland, OR).

### Quantitative RT-PCR

RNA samples were purified from the liver and small intestinal tissues in accordance with a previously described method [31]. The cDNA samples were prepared from the purified RNAs using the High Capacity cDNA Reverse Transcription Kit (Applied Biosystems, Foster city, CA, USA), and quantitative polymerase chain reaction (PCR) was performed using the SYBR Green PCR Master Mix (Applied Biosystems). Quantification was carried out using the CFX96Touch™ (Applied Biosystems). The cycling steps were as follows: 50 °C × 2 min, 95 °C × 10 min, (95 °C × 30 s, 60 °C × 30 s, 72 °C × 1 min) × 50 cycles. The primers used are summarized in Supplementary Data 4.

### Microbiome analysis

At 40 weeks post STHD-01 feeding, feces were obtained from the mice and resuspended in phosphate buffered saline (PBS; Wako, Osaka, Japan) (0.1 g/mL). The fecal suspension was smashed using the Bug Crasher (Taitec GM-01, Saitama, Japan) at maximum rotation for 10 min. The sample was on ice for 5 min and centrifuged at 2,300 × *g* at 4°C for 1 min. The DNA pellet was extracted using phenol/chloroform/isoamylalcohol (PCI; Thermo Fisher Scientific Inc., Waltham, MI). DNA was resuspended in 100 µL tris/ethylenediaminetetraacetic acid buffer (TE; Sigma) supplemented with 0.5 µL RNase A (Qiagen, Hilden, Germany). The DNA was further purified using the Template Preparation Kit (Roche, Basel, Switzerland).

The extracted DNA was measured by terminal restriction fragment length polymorphism analysis (TechnoSuruga Laboratory Co., Ltd., Shizuoka, Japan). The DNA was amplified using fluorescence-labeled primers. The amplified DNA was supplemented with the restriction enzyme BS/I (Takara Bio Inc., Tokyo, Japan) and analyzed using the ABI Prism 3130xl DNA Sequencer (Applied Biosystems) and Gene Mapper (Applied Biosystems). Cluster analysis was demonstrated using the GeneMaths program (Applied Maths, Sint-Martens-Latem, Belgium).

To measure the total number of gut bacteria in the feces, a standard curve was generated using genomic DNA from *Escherichia coli* JCM1649T. The sample DNA was amplified with primers 8F (5′ AGAGTTTGATYMTGGCTCAG 3′) and 1510R (5′ TACGGYTACCTTGTTACGACTT 3′). Quantitative PCR (qPCR) was performed using the SYBR Green PCR Master Mix (Applied Biosystems) and the CFX96Touch™ (Applied Biosystems). The cycle step was 50 °C × 2 min, 95 °C × 10 min, (95 °C × 30 s, 60 °C × 30 s, 72 °C × 1 min) × 50 cycles.

### Bile acid extraction from liver and feces

Dried feces (10 mg) were dissolved in 0.2 mL 90% ethanol and incubated at 65 °C for 1 h. The samples were then dissolved in 0.5 mL 90% ethanol. The total levels of bile acids in the plasma and feces were measured with the TBA-Test (Wako). Each liver tissue sample (0.1 g) was treated with Folch solution and homogenized. Total levels of cholesterol were measured by the T-cho E-Test (Wako). Extracted lipids were quantitatively analyzed. As a recovery standard, norcholic acid (Steraloids, Inc., Newport, RI; 1 mg/mL stock solution in 50% ethanol) was added to the ethanol. Filtrated extracts were centrifuged at 12,000 × *g* for 5 min and diluted 10 times in water. The diluted extracts were then analyzed using high-pressure liquid chromatography-tandem mass spectrometry (50 mm × 1 mm C8 column, buffered mobile phase) [32].

### HepG2 cell culture

The HepG2 cell line was obtained from the American Type Culture Collection (Manassas, VA). HepG2 cells were cultured as described previously [33]. HepG2 cells were treated with 0.6 µM or 60 µM of CA, CDCA and DCA (Sigma-Aldrich) dissolved in 0.125% dimethylsulfoxide (DMSO). After 24 h of stimulation, cells were harvested, and proteins were extracted using RIPA Buffer (Wako) supplemented with a protease inhibitor (Roche).

### Western blotting

Frozen liver tissue samples were homogenized in the T-PER solution (Thermo Fisher Scientific Inc.), supplemented with protease inhibitors (Roche), using a BioMasher (Nippi, Tokyo, Japan). Protein concentration was measured using the BCA™ Protein Assay Kit (Thermo Fisher Scientific Inc.). The homogenized tissue samples were mixed with equal parts of Laemmli Sample Buffer (Bio-Rad Laboratories Inc., Tokyo, Japan) and resolved by sodium dodecyl sulfate-polyacrylamide gel electrophoresis (SDS-PAGE; Mini-Protein^®^ TGM™ 7.5%; molecular marker, MagicMark™ XP Western Protein Standard; Invitrogen, Waltham MA). Electrophoresis was performed at 200 V for 50 min. The resolved proteins were then transferred to a nitrocellulose membrane (GE Healthcare Bioscience, Tokyo, Japan) with TE 70 semi-dry transfer unit (GE Healthcare Bioscience) at 45 mA for 90 min. After the transfer, the membranes were blocked with a blocking buffer (PBS, 0.02% Tween, and 20.5% skim milk) at 4 °C overnight. Antibodies specific for p62 (1:500, ABNOVA Products, Waltham, MI), Nrf2 (1:1000, Santa Cruz Biotechnology Inc., Santa Cruz, CA), mTOR (1:200, CST, Danvers, MA), p-mTOR (1:250, CST), Akt (1:200, CST), and p-Akt (1:300, CST) were used as primary antibodies, and a horseradish peroxidase (HRP)-conjugated anti-rabbit IgG antibody (1:5000 goat anti-rabbit IgG (H+L); Invitrogen) was used as the secondary antibody. As a control, β-actin-HRP mouse monoclonal IgG (Santa Cruz Biotechnology Inc.) was used. The membrane was reacted with ECL Western Blotting Detection Reagent (GE Healthcare Bioscience), and protein bands were visualized using an ECL mini-camera (GE Healthcare Bioscience). The density of bands was measured using ImageJ software.

### Immunohistochemistry

We tried to choose the paraffin-embedded tissue blocks containing tumoral and preferably adjacent normal liver tissue. About 5 µm. thick tissue sections were cut after that deparaffinized in xylene and rehydrated it through graded alcohols.

Then slides were rinsed with the water and immersed in TE buffer (Kanto Chemical Co., Ltd., Tokyo, Japan) at pH 8.0 in the autoclaving for antigen retrieval for 40 minutes. After washing the slides in Super Sensitive Wash Buffer (BioGenex Laboratories Inc., San Ramon, CA), we used Peroxidase Block reagent with 3% H_2_O_2_ (WAKO) to neutralize endogenous peroxidase for at-least 5 minutes. The slides were incubated with antibodies specific for p-mTOR (1:50, CST) at 4 °C overnight. After overnight the slides were washed again in Super Sensitive Wash Buffer. We incubated the slides with Histostar™ (Medical & Biological Laboratories Co., Ltd., Nagoya, Japan) for 30 minutes. Once again, washing in Super Sensitive Wash Buffer with gentle rocking followed with developing peroxidase activity with DAB working solution (BioGenex Laboratories Inc.) for 1 minutes. Then we washed the slides in water and counterstained them with Hematoxylin (Muto Pure Chemical Co., Ltd., Tokyo, Japan). After rewashing the slides in water, we finally dehydrated, cleared and mounted sections. Our slides were ready at the time for microscopic observation.

### Statistical analyses

Results were plotted as mean ± standard deviation. A one-way ANOVA followed by the Tukey’s post-hoc test was used to compare differences between multiple groups. Student’s *t*-test or Mann-Whitney *U*-test was used to compare differences between two groups. All comparisons were two-sided, and a *p*-value < 0.05 was considered significant. All statistical analyses were performed using the SPSS 22 for Windows (SPSS, IBM Japan, Tokyo, Japan).

### Consent for publication

This manuscript contains no data from patients.

### Availability of data and material

The datasets used and analyzed during the current study are available from the corresponding author on reasonable request.

## SUPPLEMENTARY MATERIALS



## Abbreviations

(NAFLD): non-alcoholic fatty liver disease
(NASH): non-alcoholic steatohepatitis
(HCC): hepatocellular carcinoma
(Mc4r): melanocortin 4 receptor
(Ltsc) 1: lacking the tumor suppressor tuberous sclerosis complex
(HFD): high-fat diet
(DMBA): 7,12-dimethylbenzanthracene
(SASP): senescence-associated secretory phenotype
(HSCs): hepatic stellate cells
(PG): prostaglandin
(MCHFD): choline-deficient high-fat diet
(LPS): lipopolysaccharide
(DSS): dextran sodium sulfate
(STHD-01): steatohepatitis-inducing HFD
(CONT): control
(SD): standard diet
(Abx): antibiotics
(T3): triiodothyronine
(T4): thyroxin
(AST): aspartate aminotransferase
(ALT): alanine aminotransferase
(Tnf): tumor nuclear factor
(Il): interleukin
(α-SMA): α-smooth muscle antigen
(Col1α1): a1 type 1 collagen
**(**Tgf)-β: transforming growth factor
(Cyp): cytochrome p450
(Asbt): apical sodium-dependent bile acid transporter
(Ost) α: organic solute transporter
(Oatp)1: organic anion-transporting polypeptide
(βMC): β-muricholic acid
(TβMC): tauro β-muricholic acid
(DCA): deoxycholic acid
(TDCA): tauro-DCA
(HDCA): and hyo-DCA
(UDCA): ursodeoxycholic acid
(TUDCA): tauro-UDCA
(KLCA): 12-Keto litho cholic acid
(CA): cholic acid
(CDCA): chenodeoxycholic acid
(mTOR): mammalian target of rapamycin
(Akt): protein kinase B
(p62): nucleoporin p62
(Nrf2): nuclear factor (erythroid-derived 2)-like 2
(FXR): farnesoid X receptor
(BSA): bovine serum albumin
(FITC): fluorescein isothiocyanate
(pDCs): plasmacytoid dendritic cells
(cDCs): conventional dendritic cells
(PCR): polymerase chain reaction
(PBS): phosphate buffered saline
(PCI): phenol/chloroform/isoamylalcohol
(TE): tris/ethylenediaminetetraacetic acid buffer
(qPCR): quantitative PCR
(DMSO): dimethylsulfoxide
(SDS-PAGE): sodium dodecyl sulfate-polyacrylamide gel electrophoresis
(HRP): horseradish peroxidase
