# Supplementary material for: Bile acid metabolism regulated by the gut microbiota promotes non-alcoholic steatohepatitis-associated hepatocellular carcinoma in mice
Source: Oncotarget. 2018 Jan 6;9(11):9925–39. doi: 10.18632/oncotarget.24066 (PMC5839411; doi:10.18632/oncotarget.24066)
Supplement: Supplementary file 1 [file oncotarget-09-9925-s001.pdf]

Bile acid metabolism regulated by the gut microbiota promotes non-alcoholic steatohepatitis-associated hepatocellular carcinoma in mice

SUPPLEMENTARY MATERIALS

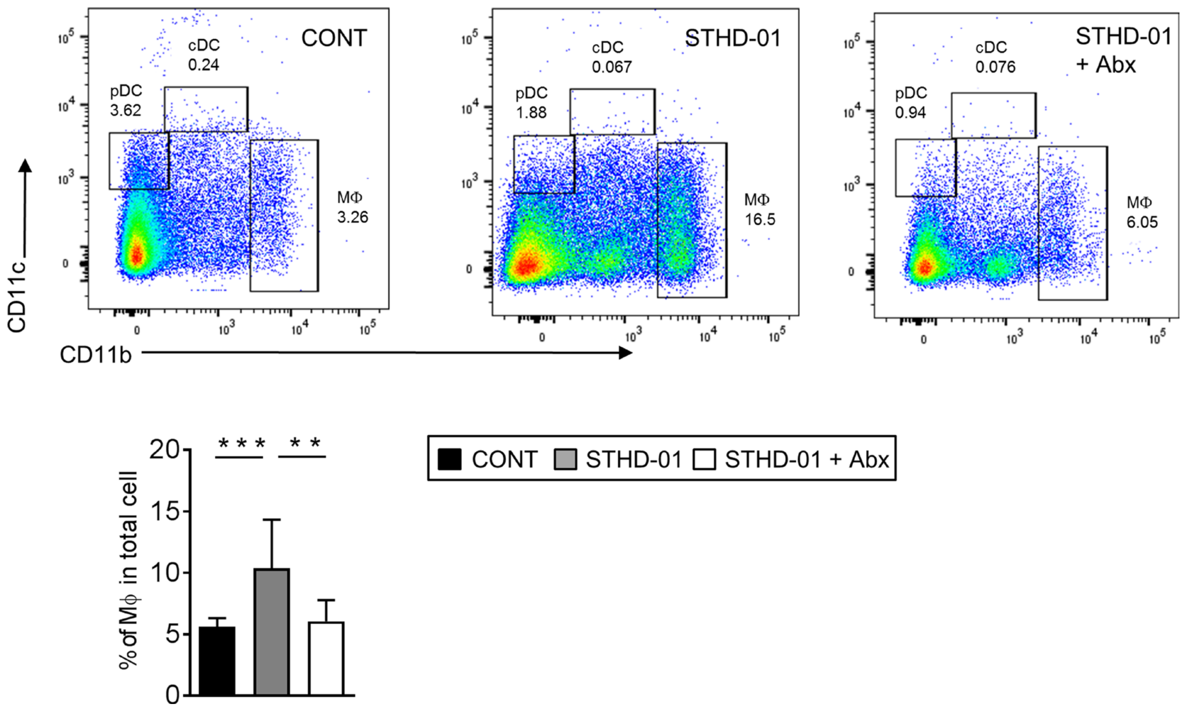

Supplementary Data 1: Infiltration of inflammatory macrophages in the liver in mice developed NASH-associated HCC. After the feeding of STHD-01 for 41 weeks, all three groups of mice were sacrificed and liver mononuclear cells were isolated. (Top) Representative fluorescence-activated cell sorting (FACS) plot for inflammatory macrophages ($CD11b^{high}CD11c^{low}$), classical DCs (cDCs; ,$CD11b^{+}CD11c^{+}$), and plasmacytoid DCs (pDCs; $CD11b^{-}CD11c^{low}$) are shown. (Bottom) The quantification of inflammatory macrophages ($CD11b^{high}CD11c^{low}$) in the liver. Data are presented as mean ± SD (n = 7). ** $p < 0.01$, *** $p < 0.001$ by Tukey's test.

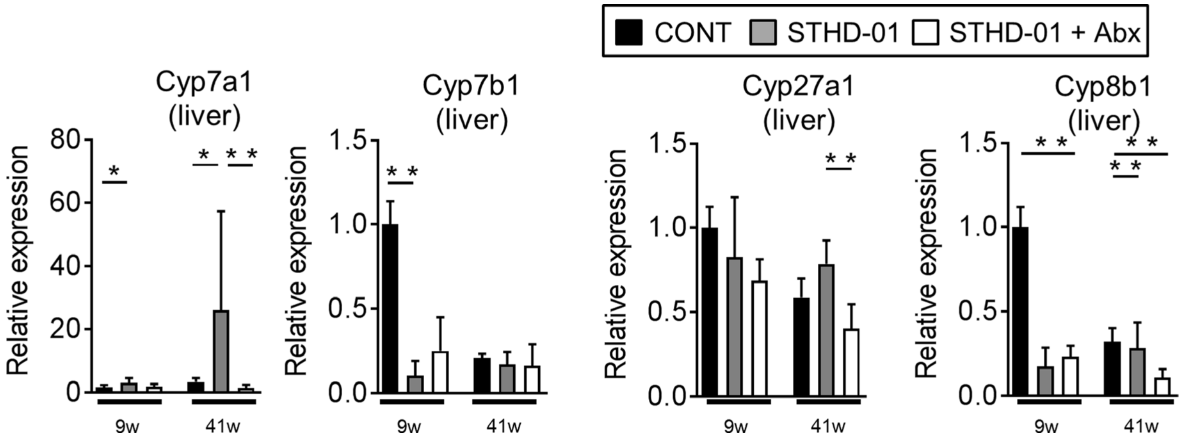

Supplementary Data 2: The expression of enzymes related to bile acid synthesis in the liver. The liver samples were harvested at 9 and 41 weeks. The expression of mRNA relative to 9 weeks control mice is shown. Data are presented as mean ± SD (CONT, $n = 5$; STHD-01, $n = 9$; STHD-01 + Abx, $n = 7$). $^{*}p < 0.05$, $^{**}p < 0.01$ by Tukey's test.

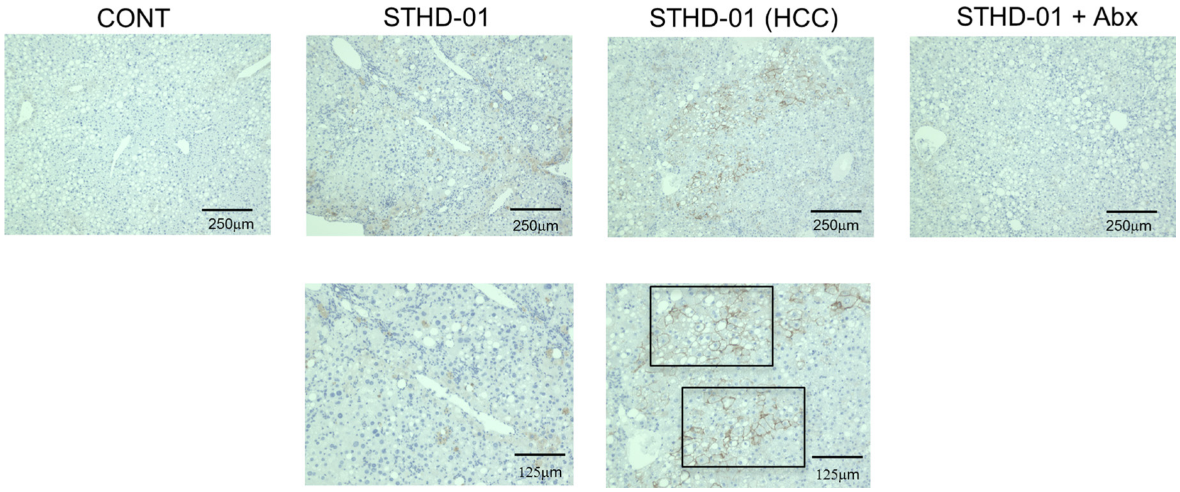

Supplementary Data 3: STHD-01 induces the activation of mTOR in the liver. The liver samples were harvested at 41 weeks. Phosphorylated form of mTOR (p-mTOR) was detected by immunostaining. Representative images are shown.

Il-1β	F	TCACAGCAGCACATCAACAA
	R	TGTCCTCATCCTGGAAGGTC
Tnf-α	F	CACGCTCTTCTGTCTACTGAACTTC
	R	ATGATCTGAGTGTGAGGGTCTGG
α-SMA	F	GACGCTGAAGTATCCGATAGAACACG
	R	CACCATCTCCAGAGTCCAGCACAAT
Col 1α1	F	GGAGGGCGAGTGCTGTGCTTT
	R	GGGACCAGGAGGACCAGGAAGT
Tgf-β	F	AGCGGACTACTATGCTAAAGAGGTCACCC
	R	CCAAGGTAACGCCAGGAATTGTTGCTATA
Cyp7a1	F	AGCAACTAAACAACCTGCCAGTACTA
	R	GTCCGGATATTCAAGGATGCA
Asbt	F	GCTTCTGTGGACTTGGCCAT
	R	TGGAGCAAGTGGTCATGCTA
Cyp7b1	F	TAGCCCTCTTTCCTCCACTCATA
	R	GAACCGATCGAACCTAAATTCCT
Cyp8b1	F	GGCTGGCTTCCTGAGCTTATT
	R	ACTTCCTGAACAGCTCATCGG
Cyp27a1	F	GCCTCACCTATGGGATCTTCA
	R	TCAAAGCCTGACGCAGATG
Oatp1	F	CAGTCTTACGAGTGTGCTCCAGAT
	R	ATGAGGAATACTGCCTCTGAAGTG
Ostα	F	TGTTCCAGGTGCTTGTCATCC
	R	CCACTGTTAGCCAAGATGGAGAA
Ostβ	F	GATGCGGCTCCTTGGAATTA
	R	GGAGGAACATGCTTGTCATGAC
Gapdh	F	GTGTCCGTCGTGGATCTGA
	R	CCTGCTTCACCACCTTCTTGA

Supplementary Data 4: The qPCR primers used in this study.
